# A First-Principles Study of the Cu-Containing β″ Precipitates in Al-Mg-Si-Cu Alloy

**DOI:** 10.3390/ma14247879

**Published:** 2021-12-19

**Authors:** Shaozhi He, Jiong Wang, Donglan Zhang, Qing Wu, Yi Kong, Yong Du

**Affiliations:** 1 State Key Laboratory of Powder Metallurgy, Central South University, Changsha 410083, China; heshaozhi@csu.edu.cn (S.H.); 203301046@csu.edu.cn (D.Z.); yikong@csu.edu.cn (Y.K.); yong-du@csu.edu.cn (Y.D.); 2 Information and Network Center, Central South University, Changsha 410083, China; wuqing@csu.edu.cn

**Keywords:** Al-Mg-Si-Cu alloys, Cu-containing β″, atomic configuration, mechanical properties, electronic structure

## Abstract

The nanostructured β″ precipitates are critical for the strength of Al-Mg-Si-(Cu) aluminum alloys. However, there are still controversial reports about the composition of Cu-containing β″ phases. In this work, first-principles calculations based on density functional theory were used to investigate the composition, mechanical properties, and electronic structure of Cu-containing β″ phases. The results predict that the Cu-containing β″ precipitates with a stoichiometry of Mg_4+*x*_Al_2−*x*_CuSi_4_ (*x* = 0, 1) are energetically favorable. As the concentration of Cu atoms increases, Cu-containing β″ phases with different compositions will appear, such as Mg_4_AlCu_2_Si_4_ and Mg_4_Cu_3_Si_4_. The replacement order of Cu atoms in β″ phases can be summarized as one Si3/Al site → two Si3/Al sites → two Si3/Al sites and one Mg1 site. The calculated elastic constants of the considered β″ phases suggest that they are all mechanically stable, and all β″ phases are ductile. When Cu atoms replace Al atoms at Si3/Al sites in β″ phases, the values of bulk modulus (*B*), shear modulus (*G*), and Young’s modulus (*E*) all increase. The calculation of the phonon spectrum shows that Mg_4+*x*_Al_2−*x*_CuSi_4_ (*x* = 0, 1) are also dynamically stable. The electronic structure analysis shows that the bond between the Si atom and the Cu atom has a covalent like property. The incorporation of the Cu atom enhances the electron interaction between the Mg2 and the Si3 atom so that the Mg2 atom also joins the Si network, which may be one of the reasons why Cu atoms increase the structure stability of the β″ phases.

## 1. Introduction

Heat treatable Al-Mg-Si(-Cu) alloys in the 6xxx series are a common category of structural materials used in the construction and transportation industries. These alloys can be customized to have a desirable combination of properties, such as good formability, high specific strength, and corrosion resistance [1,2,3]. After proper aging treatment, the strength of the alloy can be greatly improved. This is mainly due to the precipitates that can contribute to the strengthening mechanisms by hindering the dislocation movement [4,5], particle strengthening σ_p_ [6], and coherency of the particles [7]. The mechanical properties of these alloys can be greatly influenced by the composition, morphology, scale, and distribution of these solute atom nanostructures [8]. The precipitation sequence for Al-Mg-Si alloys is generally considered to be [9,10]:

$$\mathrm{SSSS}\to \;\mathrm{solute}\;\mathrm{clusters}\;\to \;\mathrm{GP}-\mathrm{zones}\to \beta^{\prime \prime }\to \beta^{\prime },U1,U2,B^{\prime }\to \beta ,\mathrm{Si}$$

The supersaturated solid solution is denoted by the abbreviation SSSS. The Guinier-Preston zones (GP-zones) were first discovered in the Al-Cu system by Guinier [11] and Preston [12]. The GP-zones mainly refer to the nanoprecipitate phases formed in the early stage of aging, which is characterized by a certain ordered structure and completely coherent with the matrix.

Among the precipitates [4,13,14,15] formed in the aged Al-Mg-Si alloys, needle-like β″ precipitate is the most effective strengthening phase [14] responsible for the peak-hardening effect [16]. The β″ phase is a metastable precipitate phase, which is semi-coherent with the Al matrix in the needle cross-section, the space group is *C*2/*m*, *a* = 15.16 Å, *b* = 4.05 Å, *c* = 6.74 Å, and *β* = 105.3° [17,18]. The monoclinic β″ phase was originally proposed to have the composition of Mg_5_Si_6_ [17]. However, according to recent experimental and theoretical studies, the composition of β″ would fluctuate around Mg_5_Al_2_Si_4_ [19,20,21,22]. Furthermore, the most recent density functional theory (DFT) calculations inferred very minor formation enthalpy differences for β″-Mg_5+*x*_Al_2−*x*_Si_4_ (−1 < *x* < 1) [21]. These results indicate that the composition of β″ phase in Al matrix may change under certain conditions. For example, the dispersed nano-precipitates can be affected by the addition of Mg and/or Si, as well as other elements like Cu [5,23,24,25,26,27]. The addition of Cu is demonstrated to increase the age-hardening response, and it promotes the generation of higher number density and smaller size precipitates [14,28,29,30,31,32]. Therefore, a certain amount of Cu is usually added into Al-Mg-Si alloys. The addition of Cu increases the complexity of the precipitation sequence [32,33]. The precipitation sequence of Al-Mg-Si-Cu alloys is reported as [34]:

$$\mathrm{SSSS}\to \;\mathrm{solute}\;\mathrm{clusters}\;\to \;\mathrm{GP}-\mathrm{zones}\to \beta^{\prime \prime },L/S/C,\mathrm{QP},\mathrm{QC}\to \beta_{}^{\prime },Q^{\prime }\to Q,\mathrm{Si}$$

Previous work used various experimental and theoretical methods to study the incorporation of Cu in β″, and analyzed the Cu atoms as foreign solute atoms in the phases [20]. Cu addition could further enhance the positive effect of pre-aging on bake hardening for Al-Mg-Si alloys [35]. It has been demonstrated by high-angle annular dark-field scanning transmission electron microscopy (HAADF-STEM) that Cu is mainly confined to the Si3/Al sites (Si or Al atoms completely occupy) of the β″ structure [26,35,36,37], which as mentioned was also supported from DFT-based calculations [38]. The β″ precipitates in Al-Mg-Si-Cu alloy were detected with an average composition of 28.6Al-38.7Mg-26.5Si-5.17Cu (at. %) using atom probe tomography (APT) and high-resolution energy-dispersive X-ray (EDX) mapping [36]. Furthermore, the addition of Cu has no effect on the type of β″ precipitate, Cu atoms incorporate in β″ and some of Mg, Si and Al in β″ unit cell are substituted by Cu atoms [39].

As mentioned above, the β″ precipitation behavior in Al-Mg-Si-Cu alloys has been investigated using various characterization methods. However, the detailed structures and stabilities are still unclear of Cu-containing β″ phases in these alloys, and these structural refinements could be supported by first-principles results [40]. In addition, we predict energy-lowering site occupations and stoichiometries of the β″ phases, where experimental information is incomplete. Understanding the structure of Cu-containing β″ precipitates is essential to elucidate the precipitation sequence in heat-treatable Al-Mg-Si (-Cu) alloys.

In the present work, first-principles calculations based on density functional theory (DFT) [41] were used to study the Cu-containing β″ phases. Based on the structural information obtained by experimental methods, first-principles atomistic calculations can provide structural, chemical, and energetic information [40]. A large number of Cu-containing β″ structures were constructed searching for possible stable configurations and structural stability, kinetic stability, and mechanical stability were also considered. Finally, the characteristics of Cu atoms occupying sites were analyzed through the electronic structure.

## 2. Materials and Methods

### 2.1. Atomic Model

**Figure 1:**
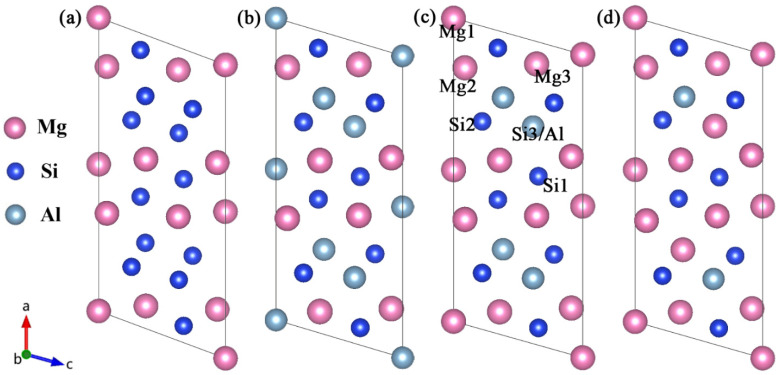
Four atomic models of the β″ available in the literature [17,21]. (**a**) Mg_5_Si_6_ from Zandbergen [17]; (**b**) Mg_4_Al_3_Si_4_ and (**c**) the Mg_5_Al_2_Si_4_ from Hasting [19]; (**d**) Mg_6_AlSi_4_ from Ehlers [21]. The relative location of each site is marked in (**c**).

For the β″ phases, the formation enthalpies and lattice parameters of Mg_4_Al_3_Si_4_, Mg_5_Al_2_Si_4_, Mg_6_AlSi_4_, and Mg_5_Si_6_ were computed for each of the models of the crystal structures available in the literature [17,18,21], allowing a critical assessment of the validity of the models. Figure 1 shows four atomic models of the β″ without Cu. The Wyckoff site information of the energetically most favorable β″-Mg_5_Al_2_Si_4_ is shown in Table 1 [18,19].

### 2.2. Computational Details

The first-principles calculations were performed utilize the plane wave pseudopotential method, as implemented in the highly efficient Vienna ab initio simulation package (VASP) [42,43], The electron-ion interactions were described through projector augmented wave (PAW) [44,45]. The exchange-correlation function were constructed by the generalized gradient approximation (GGA) of Perdew-Burke-Ernzerhof (PBE) [46]. All structures were fully relaxed with respect to atomic positions as well as all lattice parameters in order to find the lowest-energy structure. The electron wave function was expanded in plane waves up to a cutoff energy of 450 eV. The β′′phase was represented by a conventional cell with 22 atoms according to the experimental results, and 3 × 12 × 8 Γ-centered k-point meshes were employed in the Brilluion zone sampling and generated automatically by following the Monkhorst-Pack sampling scheme [47], while the 3 × 3 × 8 Γ-centered k-point meshes and 1 × 4 × 1 supercells were employed for calculation of “replacement energy” (the detailed definition is explained below). Atoms were relaxed until their residual forces converged to 0.01 eV/Å. The phonon spectra were obtained using the Phonopy package [48].

The four-parameter Birch–Murnaghan equation of state with its linear form [49] is employed to estimate the equilibrium total energy (*E*_0_), volume (*V*_0_),

(1) $$E(V)=a+bV^{-2/3}+cV^{-4/3}+dV^{-2}$$

where *a*, *b*, *c*, and *d* are fitting parameters. More details can be found in our previous work [50].

Compared with the energy of solid solution containing a Cu atom, the energy gain of the Cu atoms incorporated in β″ is referred to as “replacement energy”. In order to construct the Cu-containing β″ phases, it is necessary to determine the possible occupation sites of Cu in β″ phases. Additionally, computing the replacement energy (see Ref. [51]) can be used as a criterion for the possible occupation sites of solute atoms. There have been previous studies addressing the first-principles calculations for describing replacement energies of different sides. Since the replacement energy of Cu atoms at Mg2 and Mg3 sites were not shown in Saito’s work [38], one Cu atom was introduced into a 1 × 4 × 1 supercell and the preference of Cu atoms for each non-equivalent site in β″ was evaluated using the method described by Saito et al. [51], but with higher calculation precision.

To solve the compositional uncertainty preliminarily, the reported *C*2/*m* symmetries [18] were deliberately reduced to the level where only pairs of atoms (e.g., the two Cu atoms) were regarded as equivalent. This implies that space group *P*2/*m* was used throughout and there are 11 different sites within the unit cell. Besides, no partial occupancies were considered and vacancies were ignored. The replacement energy for Cu incorporation in β″ can be described as follows:

(2) $$\begin{array}{ll} \Delta H\left(\beta_{0}^{\prime \prime }:X\to \Xi \right)= & H\left(\beta_{0}^{\prime \prime }:3\times \{\mathrm{Al}\to \Xi \};1\times \{X\to \Xi \}\right)\\ & +H(\mathrm{fcc}\;\mathrm{Al})-H\left(\beta_{0}^{\prime \prime }:4\times \{\mathrm{Al}\to \Xi \}\right)\\ & -H(\mathrm{fcc}\;\mathrm{Al}:1\times \{X\to S\}) \end{array}$$

where *H* are the calculated enthalpy of the system, $\beta_{0}^{\prime \prime }$ are the Cu-free structure, Ξ are the sides in $\beta_{0}^{\prime \prime }$, X are the solute atoms incorporated in the precipitates, and S are substitutional sites in the Al matrix. A certain atom X incorporates on site Ξ is referred to as “{X → Ξ}”. The formation enthalpy of solid solution (SS), $\Delta H_{SS}^{\mathrm{form}}$, was used to find out the most energetically favorable configurations in the atomic models. Since there is no stable fcc structure for Mg and Si, their formation energies in relation to SS were determined as follows:

(3) $$\begin{array}{ll} \Delta H_{SS}^{\mathrm{form}\;}\left({\mathrm{Mg}}_{a}{\mathrm{Al}}_{b}{\mathrm{Cu}}_{c}{\mathrm{Si}}_{d}\right)= & E\left({\mathrm{Mg}}_{a}{\mathrm{Al}}_{b}{\mathrm{Cu}}_{c}{\mathrm{Si}}_{d}\right)-aE^{\mathrm{sub}}(\mathrm{Mg})\\ & -bE(\mathrm{Al})-cE(\mathrm{Cu})-dE^{\mathrm{sub}}(\mathrm{Si}) \end{array}$$

where *E*^sub^ (Mg) and *E*^sub^ (Si) are the enthalpies of substituting Al atoms by Mg and Si atoms, respectively. *E*^sub^ (Mg) and *E*^sub^ (Si) were calculated in a 3 × 3 × 3 Al supercell with one Mg/Si atom and 107 Al atoms with a k-point meshes of 5 × 5 × 5. The enthalpy of substituting a Mg atom was defined as:

(4) $$E^{\mathrm{sub}}(\mathrm{Mg})=E\left({\mathrm{Al}}_{107}\mathrm{Mg}\right)-107/108E(\mathrm{Al})$$

where *E* (Al) is the enthalpy of a 3 × 3 × 3 Al supercell. The definition of *E*^sub^ (Mg) was also feasible for *E*^sub^ (Si).

Finally, in order to compare the structures with different Al content, the formation enthalpy can also be expressed in kJ/mol of solute atoms, instead of kJ/mol [52]. This transformation is achieved as follows: Δ*H*_SS_ [kJ/mol solute] = Δ*H*_SS_ [kJ/mol]/(*x*_Mg_ + *x*_Si_ + *x*_Cu_), where *x*_Mg_ and *x*_Si_ and *x*_Cu_ are the atomic fractions of Mg and Si and Cu in the β″ phases Mg*_a_*Al*_b_*Cu*_c_*Si*_d_* (*a* = *x*_Mg_, *b* = *x*_Al_, *c* = *x*_Cu_, *d* = *x*_Si_). This is a common definition of formation enthalpy in the literature [9,21,52].

The elastic constant can be represented by a 6 × 6 matrix. Based on the symmetry of the crystal structure, the independent elastic constants of the monoclinic crystal are reduced to 13, as shown in Formula (5):

(5) $$C_{ij}=\left(\begin{array}{cccccc} C_{11} & C_{12} & C_{13} & 0 & C_{15} & 0\\ & C_{22} & C_{23} & 0 & C_{25} & 0\\ & & C_{33} & 0 & C_{35} & 0\\ & & & C_{44} & 0 & C_{46}\\ & & & & C_{55} & 0\\ & & & & & C_{66} \end{array}\right)$$

The stress-strain method based on the generalized Hooke’s theorem is used to calculate the elastic constants of each crystal [53]. For more detailed stress-strain method description, please refer to [54]. The relationship between elastic constant *C_ijkl_*, stress tensor *δ_kl_*, and strain tensor *δ_kl_* can be expressed as:

(6) $$\sigma_{ij}=C_{ijkl}\delta_{kl}$$

The Hill model [55] is used to further obtain the bulk modulus (*B*), shear modulus (*G*), and Youngs modulus (*E*) of the crystal through the elastic constant. The Hill model takes into account that the calculation results of the Voigt model and the Reuss model will be high and low, respectively, and take the arithmetic mean of the values of the Voigt model and the Reuss model. For monoclinic crystal structure, the formula for calculating the bulk modulus (*B*) and shear modulus (*G*) of monoclinic crystals using Voigt model and Reuss model are [56]:

(7) $$B_{V}=\frac{1}{9}\left[C_{11}+C_{22}+C_{33}+2\left(C_{12}+C_{13}+C_{23}\right)\right]$$

(8) $$\begin{array}{ll} B_{R}= & \Omega [a\left(C_{11}+C_{22}-2C_{12}\right)+b\left(2C_{12}-2C_{11}-C_{23}\right)+c\left(C_{15}-2C_{25}\right)+\\ & d\left(2C_{12}+2C_{23}-C_{13}-2C_{22}\right)+{2e\left(C_{25}-C_{15}\right)+f]}^{-1} \end{array}$$

(9) $$G_{V}=(1/15)[C_{11}+C_{22}+C_{33}+3\left(C_{44}+C_{55}+C_{66}\right)-\left(C_{12}+C_{13}+C_{23}\right)]$$

(10) $$\begin{array}{ll} G_{R}= & 15\{4[a\left(C_{11}+C_{22}+C_{12}\right)+b\left(C_{11}-C_{12}-C_{23}\right)+\\ & c\left(C_{15}+C_{25}\right)+d\left(C_{22}-C_{12}-C_{23}-C_{13}\right)+e\left(C_{15}-C_{25}\right)+f]/\Omega +\\ & 3\left[g/\Omega +\left(C_{44}+C_{66}\right)/\left(C_{44}C_{66}-C_{46}^{2}\right)\right]\}{}^{-1} \end{array}$$

wherein:

(11) $$a=C_{33}C_{55}-C_{35}^{2}$$

(12) $$b=C_{23}C_{55}-C_{25}C_{35}$$

(13) $$c=C_{13}C_{35}-C_{15}C_{33}$$

(14) $$d=C_{13}C_{55}-C_{15}C_{35}$$

(15) $$e=C_{13}C_{25}-C_{15}C_{23}$$

(16) $$\begin{array}{ll} f= & C_{11}\left(C_{22}C_{55}-C_{25}^{2}\right)-C_{12}\left(C_{12}C_{55}-C_{15}C_{25}\right)+\\ & C_{15}\left(C_{12}C_{25}-C_{15}C_{22}\right)+C_{25}\left(C_{23}C_{35}-C_{25}C_{33}\right) \end{array}$$

(17) $$g=C_{11}C_{22}C_{33}-C_{11}C_{23}^{2}-C_{22}C_{13}^{2}-C_{33}C_{12}^{2}+2C_{12}C_{13}C_{23}$$

(18) $$\begin{array}{ll} \Omega = & 2[C_{15}C_{25}\left(C_{33}C_{12}-C_{13}C_{23}\right)+C_{15}C_{35}\left(C_{22}C_{13}-C_{12}C_{23}\right)+\\ & C_{25}C_{35}\left(C_{11}C_{23}-C_{12}C_{13}\right)]-[C_{15}^{2}\left(C_{22}C_{33}-C_{23}^{2}\right)+C_{25}^{2}\left(C_{11}C_{33}-C_{13}^{2}\right)+\\ & C_{35}^{2}\left(C_{11}C_{22}-C_{12}^{2}\right)]+gC_{55} \end{array}$$

The formula for calculating the bulk modulus (*B*), shear modulus (*G*), and elastic modulus (*E*) of monoclinic crystal by Hill model [55] is:

(19) $$B_{H}=\frac{1}{2}\left(B_{V}+B_{R}\right)$$

(20) $$G_{H}=\frac{1}{2}\left(G_{V}+G_{R}\right)$$

(21) E=9BG/(3B+G)

## 3. Results and Discussion

### 3.1. Structure Stability

**Figure 2:**
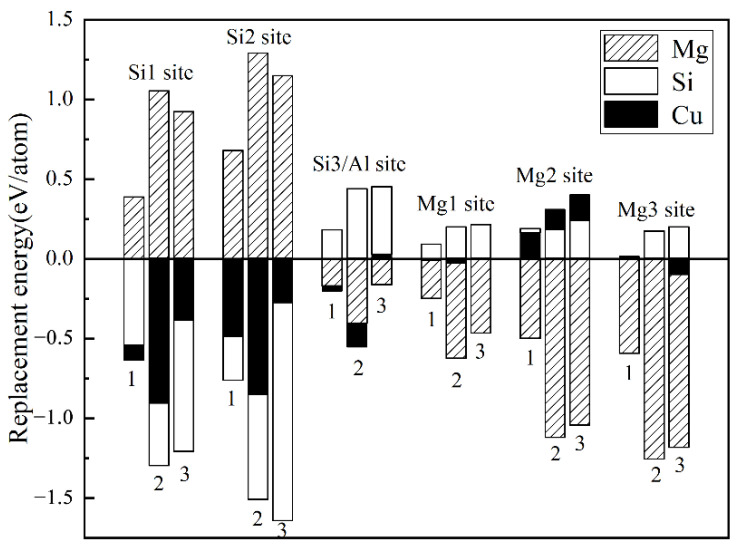
Calculated replacement energies for Cu and alternative solute atoms Mg/Si on the different sites of three different β″ configurations. 1: Mg_4_Al_3_Si_4_, 2: Mg_5_Al_2_Si_4_, and 3: Mg_6_AlSi_4_. The position of each column represents a different position in a different configuration. Cu, Mg, and Si replacement energies are labelled with black, shaded, and white bars, respectively.

**Figure 3:**
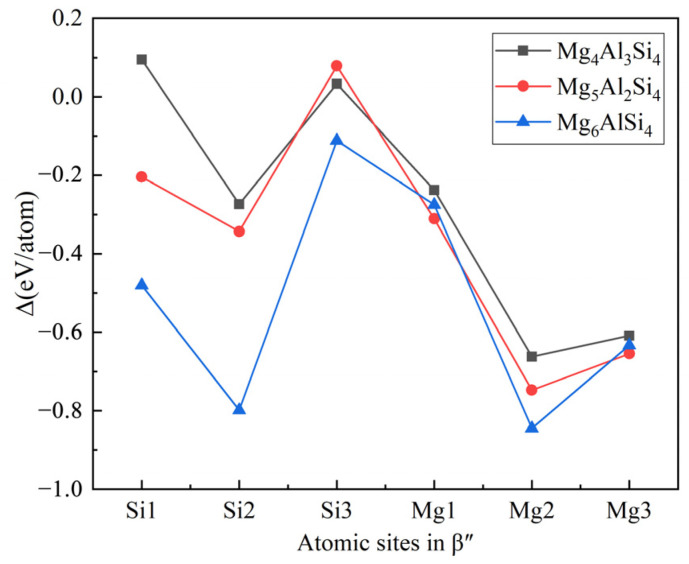
The competitiveness (Δ values) of Cu atoms at different sites in different β″ configurations. The black square, red circle, and blue triangle represent Cu atoms in the Mg_4_Al_3_Si_4_, Mg_5_Al_2_Si_4_, and Mg_6_AlSi_4_ configurations, respectively.

The replacement energy is shown in Figure 2 and alternative solute atoms Mg/Si were incorporated for comparison with Cu at different sites. In order to more intuitively express the competitive occupation sites of Cu atoms, the variable Δ is introduced and the Δ values of Cu atoms at different sites in different β″ configurations are shown in Figure 3. The Δ represents the “competitiveness” between Cu atoms and other solute atoms at each site, it is the difference between the lowest replacement energy of Mg/Si solute atoms and the replacement energy of Cu atoms. The larger the value of Δ, the more likely the Cu atom will occupy the site. Consequently, due to the low Cu occupancy in β″, only three designated Cu sites (Si1, Si3, Mg1, see Figure 1c) were allowed to host Cu atoms according to the relative value of replacement energy (refer to Figure 2). This conclusion is consistent with previous research [38].

**Figure 4:**
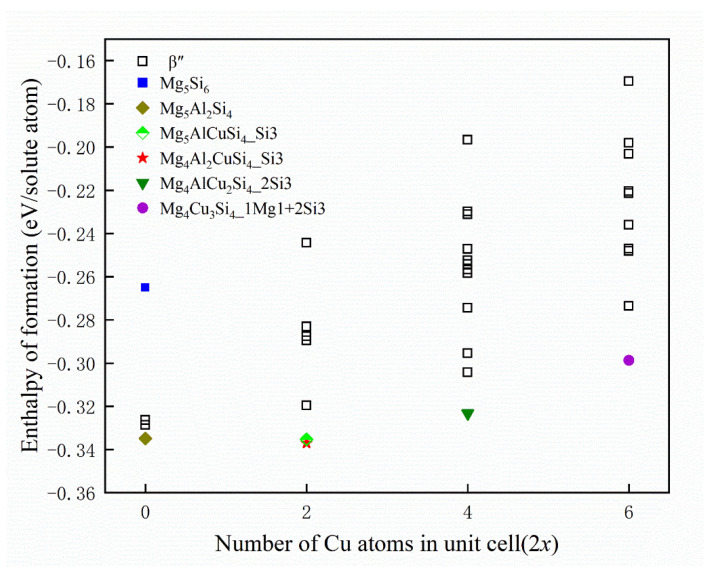
Formation enthalpies of calculated β″ phases. When Cu atoms occupy *x* sites the number of Cu atoms in unit cell is corresponding to 2*x*. The lower energy configuration is marked with a specific shape, and Mg_5_Si_6_ is also marked as a reference. 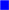 represents the Mg_5_Si_6_, 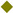 represents the Mg_5_Al_2_Si_4_, 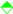 represents the Mg_5_AlCuSi_4_ where Cu atoms occupy a Si3 site, 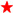 represents the Mg_4_Al_2_CuSi_4_ where Cu atoms occupy a Si3 site, 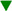 represents the Mg_4_AlCu_2_Si_4_ where Cu atoms occupy two Si3 sites, and 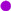 represents the Mg_4_Cu_3_Si_4_ where Cu atoms occupy a Mg1 site and two Si3 sites.

**Figure 5:**
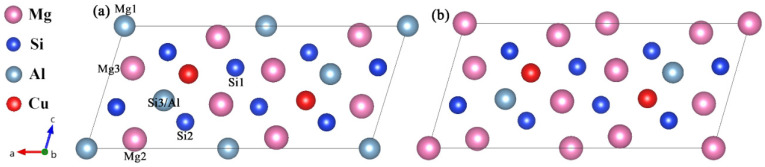
The well-defined Mg_4+*x*_Al_2−*x*_CuSi_4_ (*x* = 0, 1) unit cell that sole minimization of the β″ phase formation enthalpy supports. (**a**) Mg_4_Al_2_CuSi_4_; (**b**) Mg_5_AlCuSi_4_. The relative location of each site is marked in (**a**).

For checking the reliability of the calculations, Table 2 displays the structural parameters for selected β″ configuration without Cu atom, along with the results of earlier theoretical and experimental studies of β″. Available calculation results of formation enthalpies are shown in Table 3. The formation enthalpies of the 33 possible unit cells have been plotted in Figure 4, including the configuration without Cu atom. Since the given formation enthalpy of per solute atom (eV/solute atom) essentially presents the solute chemical potentials, the zero-temperature convex hull can be constructed to deduce the precipitation order of the system [57]. It can be seen that Cu occupying one column of each Si3 column pair is found to be the energetically most favorable option for the set of Mg_4_Al_2_CuSi_4_ compositions. While the formation enthalpy of Mg_4_Al_2_CuSi_4_ is −0.337 eV/solute atom, the formation enthalpy of Mg_5_AlCuSi_4_ is −0.335 eV/solute atom, which is similar to that of Mg_4_Al_2_CuSi_4_. This is consistent with the observed in previous experiments that Cu atoms mainly occupy Si3 sites [36]. The energy gained when replacing Mg/Si/Al with at the Wyckoff sites is clearly varying with *x*. When Cu atoms occupy two sites (that is, *x* = 2), Mg_4_AlCu_2_Si_4_ is the energetically most favorable phase, and Cu atoms occupy two Si3 columns. When Cu atoms occupy three sites, Mg_4_Cu_3_Si_4_ is the most stable structure, in which Cu atoms occupy one Mg1 site and two Si3 sites, which is consistent with experimental observations [36]. The results show that stoichiometry of Cu-containing β″ phase is suggested as Mg_4_Al_3−*x*_Cu*_x_*Si_4_ (1 ≤ *x* ≤ 3). Since the formation enthalpy of Mg_5_AlCuSi_4_ is very close to that of Mg_4_Al_2_CuSi_4_, it can also be taken into account. This result emphasizes the possibility of fluctuations between various compositions as a function of the local alloying element concentration for the physical system during precipitated phases growth. Then the structural parameters of low energy configurations from Figure 4 also have been displayed in Table 2. As discussed above, sole minimization of the β″ phase formation enthalpy supports the well-defined Mg_4+*x*_Al_2−*x*_CuSi_4_ (*x* = 0, 1) unit cell shown in Figure 5.

### 3.2. Elastic Properties

**Figure 6:**
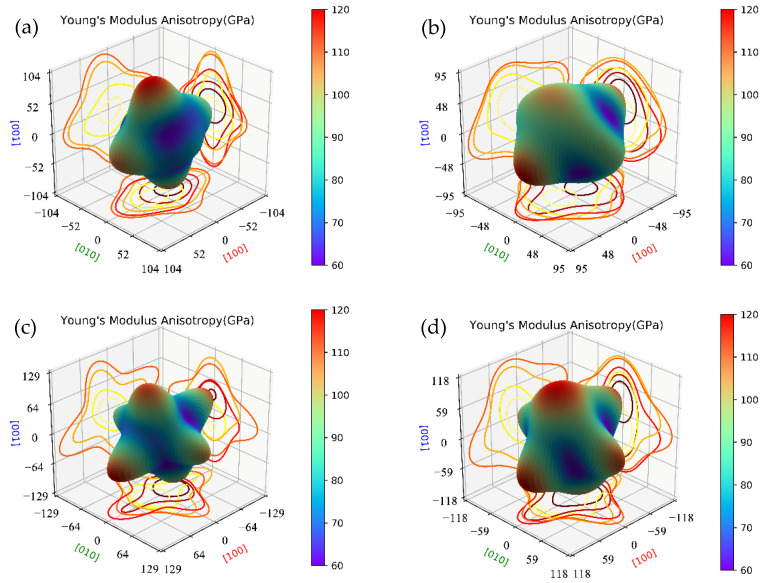
The Young’s modulus anisotropies three-dimensional map of β″ phases. (**a**) Mg_4_Al_3_Si_4_; (**b**) Mg_5_Al_2_Si_4_; (**c**) Mg_4_Al_2_CuSi_4_; (**d**) Mg_5_AlCuSi_4_.

Here, we compare the mechanical properties of β″ with or without Cu atoms. The elastic constants of key β″ phases that are most likely to precipitate during aging were calculated by using fully relaxed crystal structures, and the results are listed in Table 4. According to the Born stability criterion [61], the elastic constants of Mg_4_Al_2_CuSi_4_ and Mg_5_AlCuSi_4_ all meet the stability criteria of monoclinic crystals. This further supports the stability of Mg_4+*x*_Al_2__−_*_x_*CuSi_4_ (*x* = 0, 1) obtained from the formation enthalpy. The elastic constants *C*_11_, *C*_22_, and *C*_33_ are much greater than the other elastic constants in all calculated β″ phases, resulting in an obvious elastic anisotropy. In order to understand the anisotropic characteristics of these precipitation phases, the Young’s modulus anisotropies are evaluated by three-dimensional map as shown in Figure 6. Comparing Figure 6a and c, it can be seen that after Cu atoms substituted Al atoms on the Si3/Al sites, the Young’s modulus (*E*) anisotropy increases significantly; similar results are also shown in Figure 6b,d. This phenomenon indicates that the growth rate of the Cu-containing β″ phases may be faster than that of the β″ without Cu. It is consistent with the previous study that Cu can accelerates the age-hardening response [14,28,30].

Based on the elastic constants in Table 4, the bulk modulus (*B*), shear modulus (*G*), and Young’s modulus (*E*) of polycrystalline are calculated by the Hill model [55], and the results are listed in Table 5. Comparing the values of *E*, *G*, and *B* of Mg_4_Al_3_Si_4_ and Mg_4_Al_2_CuSi_4_, it can be seen that the values of *E*, *G*, and *B* of β″ with Cu atoms are higher than that of β″ without Cu atoms. This relationship is also shown between Mg_5_Al_2_Si_4_ and Mg_5_AlCuSi_4_. In general, the Young’s modulus (*E*) can be used to measure the stiffness of the material. The stiffness of the material is greater with the increasing of Young’s modulus (*E*) [64]. It is obvious that the stiffness is enhanced after Cu incorporate into Si3 sites. Pugh [65] proposes using the ratio of the bulk and shear modulus, *B*/*G*, to predict brittle or ductile behavior of materials. According to the Pugh criterion, if *B*/*G* is more than 1.75, ductile behavior is expected; otherwise, the material would be brittle. From Table 4, the *B*/*G* values of calculated β″ phases are all larger than 1.75, therefore, all the compounds of β″ phase are ductile with or without Cu atoms and the ductility decreases after Cu atoms incorporate into β″. In addition, Poisson’s ratio *v* has been used to measure the shear stability of the lattice, which usually ranges from −1 to 0.5. The smaller the value, the stronger the ability of the crystal to maintain stability during shear deformation [66]. The value of Poisson’s ratio *v* > 0.26 means the ductility of the materials, and the Poisson’s ratio of metals is usually 0.25< *v* < 0.35 [67]. As one can see, all β″ configurations show ductility with minor differences. It is consistent with the conclusion based on Pugh criterion.

### 3.3. Phonon Spectra

**Figure 7:**
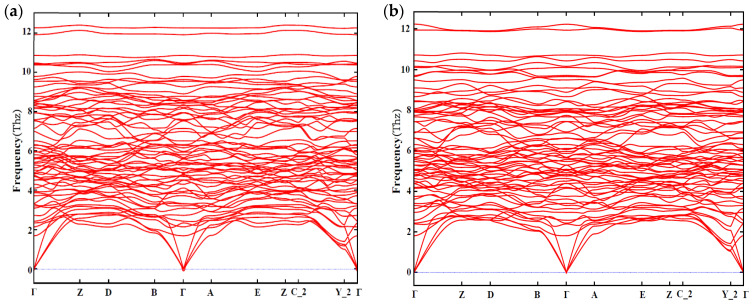
The phonon spectrum along a highly symmetric K-points path of (**a**) Mg_4_Al_2_CuSi_4_; (**b**) Mg_5_AlCuSi_4_.

In addition, the dynamic stability is also taken into account. The phonon spectra of Mg_4_Al_2_CuSi_4_ and Mg_5_AlCuSi_4_ are shown in Figure 7. From Figure 7, one can see that there is no virtual frequency of configuration Mg_4_Al_2_CuSi_4_ and Mg_5_AlCuSi_4_, which is generally considered to be dynamically stable.

### 3.4. Electronic Structure

**Figure 8:**
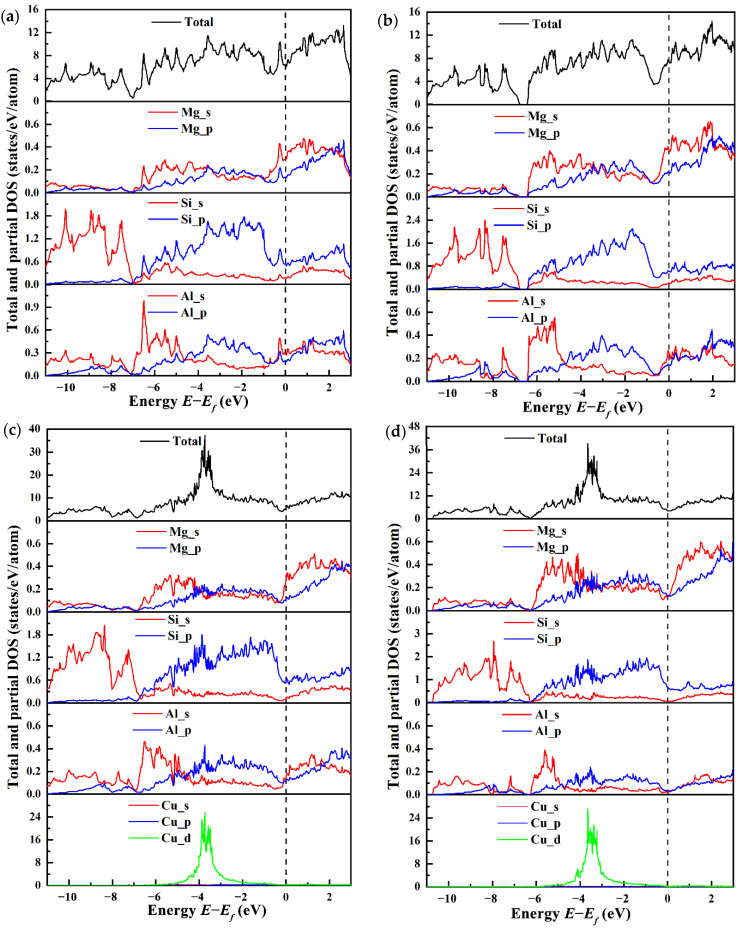
The total and partial electronic density of states (PDOSs and TDOSs) for four β″ type compounds. (**a**) Mg_4_Al_3_Si_4_; (**b**) Mg_5_Al_2_Si_4_; (**c**) Mg_4_Al_2_CuSi_4_; (**d**) Mg_5_AlCuSi_4_.

The total and partial electronic density of states (TDOSs and PDOSs) for four types of β″ configurations are calculated to explore the influence mechanism of electronic interaction on structural stability and mechanical properties, as shown in Figure 8, with the Fermi level set to zero. It is evident that incorporating Cu does not change the metallic characteristic of the β″ phase due to the finite DOS at the Fermi level. At the Fermi level, the TDOS for four types of β″ configurations at the Fermi level varies. The greatest *n* (*E_f_*) is 7.41 states/eV/cell in Mg_5_Al_2_Si_4_, followed by 6.40 states/eV/cell in Mg_4_Al_3_Si_4_, 5.27 states/eV/cell in Mg_4_Al_2_CuSi_4_, and 4.18 states/eV/cell in Mg_5_AlCuSi_4_. This indicates that the Cu-containing β″ phases have a smaller *n* (*E_f_*). In general, a smaller pseudo gap value *n* (*E_f_*) corresponds to a more stable structure [68]. This indicates that Mg_4+*x*_Al_2−*x*_CuSi_4_ (*x* = 0, 1) are more stable than the β″ phases without Cu. The Si-s (range from around 11 eV to 7 eV) and Si-p states (from around 7 eV to the Fermi level) dominate the TDOS of Mg_4_Al_3_Si_4_ and Mg_5_Al_2_Si_4_ below the Fermi level. In between (ranging from about −7 eV up to −4 eV) regimes, a mixture of s and p character exists, indicating strong hybridization. Especially from −7 eV to −5 eV, the shapes of Si-s and Si-p are very similar, indicating that there is a strong interaction between Si atoms. This may be the origin for the formation of the Si-network; the Si-network acts as a stable skeleton of these phases [32,69]. One can see that Mg-s/Al-s and Si-p in the range from −7 to −4 eV, originating mainly from the s-p hybridization of Si atoms and Mg/Al atoms. The s-states and p-states of Al, Mg, and Si are strongly hybridized above the Fermi level. From Figure 8, it should be noted that, below the Fermi level, the Cu-d state is formed. The s/p orbitals of Mg, Si, and Al all interact with the Cu-d state, and there is obvious electron transfer. The Si-p orbital and the Cu-d orbital are hybridized to form a covalent like bonding, and more electrons are transferred to the new orbital formed by the p-d hybridization.

**Figure 9:**
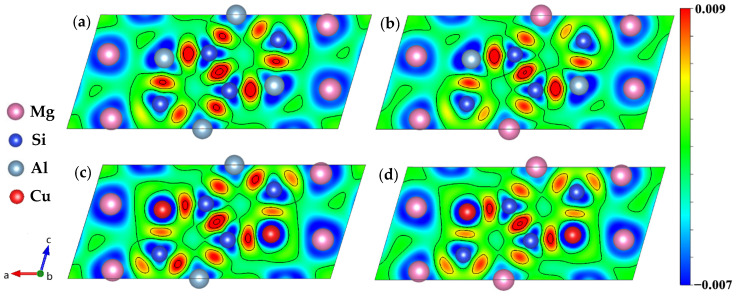
Charge density difference of four calculated β″ phases. (**a**) Mg_4_Al_3_Si_4_; (**b**) Mg_5_Al2Si4; (**c**) Mg_4_Al_2_CuSi_4_; (**d**) Mg_5_AlCuSi_4_.

In order to gain a better understanding of the electronic structure of the studied system, the charge density distributions were used as an additional method. The charge-density difference between the (DFT) converged charge density and the isolated atomic charge densities were employed. Figure 9 shows the charge density difference contour plot for the (010) plane to analyze the interaction between Al, Mg, Si, and Cu atoms for the β″ phases. Here we clearly see that there has indeed been a transfer of charge to all the Si–Si bond regions, it is consistent with the analysis by Derlet et al. [69]. A dominant feature of Figure 9a,b is the concentration of charge between the Si1-S3/Al-Si2-Mg1-Si1 nearest neighbors, and to a lesser extent, between the Si3 and Mg1 nearest neighbors, indicating that covalency plays a role in this system, which was also reported in previous research [70]. Meanwhile, the charge distribution looks like a “charge loop”, which can lead to the formation of an “Si network”. Strong covalent bonds network can significantly increase the structural stability of β″ phases. Such a charge transfer to the bonding regions originates from the core regions of both atoms on the Mg and Si sites, in addition to the homogeneous interstitial region between the Mg atoms. The depletion of charge from the Mg3 sites indicates that for this system both metallicity and covalency are present in the bonding. Moreover, the charge transfer density between the Si3 and Si2 sites is slightly decreased, and the charge transfer density between the Mg2 and Si2 sites is increased, indicating the bonds between atoms on Mg2 and Si2 sites are covalent. As shown in Figure 9, the charge ionization of all Mg3 sites is strong, and when the Cu atom on the Si3 site charge ionization becomes stronger, it means both Mg and Cu valence electron are delocalized. The difference is that Mg uniformly provides charges to the surroundings to form a metallic environment [69], while the charges of Cu atoms are delocalized toward Si atoms in unit cells, forming a directional covalent like bond.

According to the analysis of the thermodynamic results of replacement energies and formation enthalpies in Section 3.1 “Structure stability”, the stoichiometry of Cu-containing β″ phases in the precipitation sequence and the sequence of Cu atoms substituting sites in the β″ phases can be inferred. For the stable phases determined from the thermodynamics, the elastic properties of β″ phases with and without Cu were calculated in Section 3.2 “Elastic properties”, further supporting the proposed stoichiometry. Besides, the “Phonon spectra” study in Section 3.3 shows that they are also dynamically stable. In summary, the proposed compositions Mg_4_Al_3−*x*_Cu_x_Si_4_ (1 ≤ *x* ≤ 3) are reasonable, which is consistent with the results observed in the experiment [36]. In the Section 3.4 “Electronic structure”, the origin for the stability of the Cu-containing β″ phases is analyzed from the perspective of electron interaction.

## 4. Conclusions

(1) The calculation of the formation enthalpies of 33 Cu-containing β″ phases shows that the replacement order of Cu atoms in β″ phases can be summarized as one Si3/Al site → two Si3/Al sites → two Si3/Al sites and one Mg1 site.
(2) The Cu atoms strongly favor occupying one of each pair of Si3/Al sites and the most stable Cu-containing β″ phases were expected to have a stoichiometry of Mg_4+*x*_Al_2−*x*_CuSi_4_ (*x* = 0, 1). In addition, taking into account the change of Cu content in β″ phases, the stoichiometry of Mg_4_Al_3−*x*_Cu_x_Si_4_ (1 ≤ *x* ≤ 3) may precipitate.
(3) The calculated mechanical properties show that all calculated β″ phases are mechanically stable. The incorporation of Cu atoms improves the values of bulk modulus (*B)*, shear modulus (*G),* and Young’s modulus *(E)* of β″, respectively, and all β″ phases calculated show ductile behavior. Furthermore, the calculation of the phonon spectra shows that Mg_4+*x*_Al_2−*x*_CuSi_4_ (*x* = 0, 1) are dynamically stable.
(4) The electronic structure results shows that the Cu atom will join the Si network, and the bond between the Si atom and the Cu atom has the covalent property. The incorporation of Cu atom increases the electron interaction between the Mg2 and the Si3 atom, which may be one of the reasons why the incorporation of Cu atom increases the stability of the β″ phase structure.

## Figures and Tables

**Table 1:** Wyckoff site information (*x*, *y*, *z*) in the β″-Mg_5_Al_2_Si_4_ phase [18,19]; atomic configuration is shown schematically in Figure 1c.

Site	Occupation	*x*	*y*	*z*
Mg1	2*a*	0	0	0
Mg2	4*i*	0.3419	0	0.099
Mg3	4*i*	0.4225	0	0.659
Si1	4*i*	0.0501	0	0.678
Si2	4*i*	0.1876	0	0.225
Si3/Al	4*i*	0.2213	0	0.618

**Table 2:** First-principles (VASP-GGA) and experimental lattice parameters of β″ phases of Al-Mg-Si-(Cu) system. For the Cu-containing β″ phases, only the most stable crystal structures under different Cu concentrations are listed.

Configurations	*a* (Å)	*b* (Å)	*c* (Å)	*β* (°)	Ref.
Mg_5_Si_6_	15.12	4.04	6.99	110.6	
Mg_5_Si_6_ (exp.)	15.16 ± 0.02	4.05	6.74 ± 0.02	105.3 ± 0.5	[17]
Mg_5_Si_6_ (GGA)	15.11	4.080	6.932	110.4	[21]
Mg_5_Si_6_ (GGA)	15.13	4.05	6.96	110	[58]
Mg_5_Si_6_ (GGA)	15.12	4.084	6.928	110.5	[59]
Mg_5_Si_6_ (GGA)	15.14	4.05	6.94	110	[60]
Mg_4_Al_3_Si_4_	15.05	4.16	6.59	106.6	
Mg_4_Al_3_Si_4_ (GGA)	15.11	4.131	6.615	106.6	[21]
Mg_5_Al_2_Si_4_	15.36	4.05	6.79	105.7	
Mg_5_Al_2_Si_4_ (GGA)	15.32	4.075	6.778	105.9	[21]
Mg_5_Al_2_Si_4_ (GGA)	15.50	4.05	6.74	106	[19]
Mg_6_AlSi_4_	15.63	4.06	6.82	105.9	
Mg_6_AlSi_4_ (GGA)	15.59	4.069	6.830	106.1	[21]
Mg_4_Al_2_CuSi_4_	14.78	4.02	6.69	107.3	
Mg_5_AlCuSi_4_	15.08	3.95	6.86	106.2	
Mg_4_AlCu_2_Si_4_	14.46	4.03	6.68	109.2	
Mg_4_Cu_3_Si_4_	14.17	4.11	6.37	107.5	

**Table 3:** Formation enthalpies of β″ phases with different configurations in this work. The Cu occupied sites and its number are also listed in detail.

Configurations	Cu Occupied Sites	*x*_Mg_/(*x*_Mg_ + *x*_Si_)	*x*_Cu_	Δ*E*_β″_ (eV/Solute Atom)
Mg_5_Si_6_	-	0.45	0.00	−0.2650
Mg_5_Si_6_ [21]	-	0.45	0.00	−0.2665
Mg_4_Al_3_Si_4_	-	0.50	0.00	−0.3264
Mg_5_Al_2_Si_4_	-	0.56	0.00	−0.3348
Mg_5_Al_2_Si_4_ [21]	-	0.56	0.00	−0.3456
Mg_6_AlSi_4_	-	0.60	0.00	−0.3286
Mg_6_AlSi_4_ [21]	-	0.60	0.00	−0.3380
Mg_4_Al_3_CuSi_3_	1 Si1	0.57	0.09	−0.2896
Mg_4_Al_2_CuSi_4_	1 Si3/Al	0.50	0.09	−0.3370
Mg_4_Al_2_CuSi_4_	1 Mg1	0.50	0.09	−0.3196
Mg_4_Al_3_Cu_2_Si_2_	2 Si1	0.67	0.18	−0.2525
Mg_4_AlCu_2_Si_4_	2 Si3/Al	0.50	0.18	−0.3232
Mg_4_Al_2_Cu_2_Si_3_	1 Si1 and 1 Si3	0.57	0.18	−0.2567
Mg_4_Al_2_Cu_2_Si_3_	1 Si1 and 1 Mg1	0.57	0.18	−0.2583
Mg_4_AlCu_2_Si_4_	1 Si3/Al and 1 Mg1	0.50	0.18	−0.3043
Mg_4_Al_2_Cu_3_Si_2_	2 Si1 and 1 Si3/Al	0.67	0.27	−0.2205
Mg_4_AlCu_3_Si_3_	1 Si1 and 2 Si3/Al	0.57	0.27	−0.2737
Mg_4_Al_2_Cu_3_Si_2_	2 Si1/Al and 1 Mg1	0.67	0.27	−0.2215
Mg_4_Cu_3_Si_4_	2 Si3/Al and 1 Mg1	0.50	0.27	−0.2988
Mg_4_AlCu_3_Si_3_	1 Si1 and 1 Si3/Al and 1 Mg1	0.57	0.27	−0.2482
Mg_5_Al_2_CuSi_3_	1 Si1	0.63	0.09	−0.2831
Mg_5_AlCuSi_4_	1 Si3/Al	0.56	0.09	−0.3352
Mg_5_Al_2_Cu_2_Si_2_	2 Si1	0.71	0.18	−0.2298
Mg_5_Cu_2_Si_4_	2 Si3/Al	0.56	0.18	−0.2955
Mg_5_AlCu_2_Si_3_	1 Si1 and 1 Si3	0.63	0.18	−0.2542
Mg_5_AlCu_3_Si_2_	2 Si1 and 1 Si3	0.71	0.27	−0.2033
Mg_5_Cu_3_Si_3_	1 Si1 and 2 Si3	0.63	0.27	−0.2471
Mg_6_AlCuSi_3_	1 Si1	0.67	0.09	−0.2444
Mg_6_CuSi_4_	1 Si3/Al	0.60	0.09	−0.2876
Mg_6_AlCu_2_Si_2_	2 Si1	0.75	0.18	−0.1967
Mg_6_Cu_2_Si_3_	1 Si1 and 1 Si3	0.67	0.18	−0.2311
Mg_5_AlCu_2_Si_3_	1 Si1 and 1 Mg1	0.63	0.18	−0.2472
Mg_5_Cu_2_Si_4_	1 Si3/Al and 1 Mg1	0.56	0.18	−0.2744
Mg_5_AlCu_3_Si_2_	2 Si1 and 1 Mg1	0.71	0.27	−0.1981
Mg_6_Cu_3_Si_2_	2 Si1 and 1 Si3	0.75	0.27	−0.1696
Mg_5_Cu_3_Si_3_	1 Si1 and 1 Si3 and 1 Mg1	0.63	0.27	−0.2361

**Table 4:** Calculated single crystal elastic stiffness constants (*C_ij_*′s) of the reported β″ phases and energy favorable Cu-containing β″ phases.

Configuration	*C*_11_	*C*_12_	*C*_13_	*C*_15_	*C*_22_	*C*_23_	*C*_25_	*C*_33_	*C*_35_	*C*_44_	*C*_46_	*C*_55_	*C*_66_
Mg_5_Si_6_	110	42	42	−3	103	49	4	94	11	19	5	17	25
Mg_5_Si_6_ [62]	106	49	50	−11	90	46	6	88	9	17	1	33	30
Mg_5_Si_6_ [63]	98	50	48	8	84	46	6	88	5.4	22	−10	29	51
Mg_4_Al_3_Si_4_	119	52	35	−3	99	47	3	122	10	19	−1	29	20
Mg_4_Al_3_Si_4_ [62]	114	46	48	−4	104	49	6	104	7	21	0	34	23
Mg_4_Al_3_Si_4_ [63]	107	47	48	9	97	48	6	97	9	26	6	36	46
Mg_5_Al_2_Si_4_	111	38	44	−4	102	46	3	106	7	25	4	31	25
Mg_5_Al_2_Si_4_ [62]	108	42	48	−3	95	46	5	100	3	23	4	33	27
Mg_5_Al_2_Si_4_ [63]	107	40	46	−13	95	43	4	99	12	27	5	36	49
Mg_6_AlSi_4_	121	28	40	−5	125	28	2	117	6	28	4	35	21
Mg_4_Al_2_CuSi_4_	136	44	48	−13	133	43	9	130	14	25	3	35	23
Mg_5_AlCuSi_4_	127	41	46	−9	128	32	5	131	6	31	4	38	22
Mg_4_AlCu_2_Si_4_	128	44	70	−3	136	53	6	103	10	28	7	32	22
Mg_4_Cu_3_Si_4_	128	47	75	7	153	43	3	115	−6	20	3	51	26

**Table 5:** Calculated mechanic properties of the reported β″ phases and energy favorable Cu-containing β″ phases.

Configurations	*B* (GPa)	*G* (GPa)	*E* (GPa)	*B*/*G*	*ν*
Mg_5_Si_6_	62	22	60	2.77	0.34
Mg_5_Si_6_ [63]	62	-	-	-	-
Mg_4_Al_3_Si_4_	67	26	69	2.57	0.33
Mg_4_Al_3_Si_4_ [63]	64	-	-	-	-
Mg_5_Al_2_Si_4_	63	28	74	2.23	0.30
Mg_5_Al_2_Si_4_ [63]	61	-	-	-	-
Mg_6_AlSi_4_	62	33	84	1.87	0.27
Mg_4_Al_2_CuSi_4_	72	32	84	2.26	0.31
Mg_5_AlCuSi_4_	69	34	88	2.01	0.29
Mg_4_AlCu_2_Si_4_	76	28	74	2.73	0.34
Mg_4_Cu_3_Si_4_	81	32	84	2.54	0.33

## Data Availability

Data are contained within the article.
